# Nonsurgical periodontal treatment and prosthetic rehabilitation of a renal transplant patient with gingival enlargement: a case report with 2-year follow-up

**DOI:** 10.1186/s12903-018-0607-2

**Published:** 2018-08-20

**Authors:** Chi-Ching Chang, Tai-Min Lin, Chiu-Po Chan, Whei-Lin Pan

**Affiliations:** 1 Department of Periodontics, Taipei Chang Gung Memorial Hospital, Graduate Institute of Dental and Craniofacial Science, Chang Gung University, 6F., No.199, Dunhua N. Rd., Songshan Dist., Taipei City, 105 Taiwan; 2 Department of Prosthodontics, Taipei Chang Gung Memorial Hospital, Graduate Institute of Dental and Craniofacial Science, Chang Gung University, 6F., No.199, Dunhua N. Rd., Songshan Dist., Taipei City, 105 Taiwan

**Keywords:** Nonsurgical treatment, Gingival enlargement, Cyclosporine, Pathological tooth migration

## Abstract

**Background:**

Drug-induced gingival enlargement is a common condition which can be observed in patients taking immunosuppressive medications following organ transplant surgery. The disfiguring excessive tissue often hinders proper oral hygiene practices, therefore accompanied by periodontitis, tooth mobility, and even pathological tooth migration in extreme cases. This case report presents a conservative treatment protocol for a patient with the aforementioned conditions involving neither surgical nor orthodontic intervention. Few related studies have reported such a noninvasive protocol for managing these kinds of conditions.

**Case presentation:**

A 51-year-old woman presented with bleeding gingiva, mobile teeth and complained of chewing difficulties. She had undergone renal transplant surgery 16 years prior to this dental visit and had been taking immunosuppressive drugs including cyclosporine ever since. After clinical and radiographic examinations, the patient was diagnosed with drug-induced gingival enlargement, pathological tooth migration, severe periodontitis, and missing teeth. Through careful and meticulous nonsurgical debridement, oral hygiene instruction, tooth extraction, and occlusal adjustment, the patient’s periodontium was restored to a healthy state without surgical intervention. Moreover, the patient’s chewing function was restored by means of removable partial dentures. Good adaptation of prostheses and satisfaction with overall treatment outcomes were reported.

**Conclusions:**

Through proper diagnosis, treatment, and with good patient cooperation, complex systemic and dental problems can be managed conservatively without invasive surgeries to attain a stable periodontium and eventually, occlusal function could be restored.

## Background

The kidneys are vital organs responsible for multiple functions in the human body including metabolic waste excretion, water and electrolyte balance, hormone production, and body fluid osmolality regulation. In cases of conditions such as diabetes and hypertension or under the influence of various drugs, the kidneys might gradually lose their function, which can subsequently result in chronic kidney disease (CKD). As CKD progress to stage 5, which is also called end-stage renal disease (ESRD) or kidney failure, dialysis or transplantation would be inevitable [1].

Renal transplantation is a reliable treatment option for patients with ESRD, but these patients would have to take immunosuppressive drugs throughout their lives. Cyclosporine is a widely used immunosuppressive agent, and gingival enlargement is a well-documented side effect of this drug [2, 3]. Gingival enlargement refers to fibrotic and sometimes lobulated growth disfigurement in the oral soft tissue, usually found at the anterior facial interdental papillae. The prevalence of gingival enlargement with respect to the immunosuppressive drug cyclosporine is 25–30% in adults and over 70% in children [4]. Patients with gingival enlargement often hinders proper oral hygiene practice [5] and may induce periodontal diseases. Based on the increasing number of transplantations being performed [6], this condition is increasingly seen in dental offices; treating such patients is often challenging [7].

Pathological tooth migration is a common complication of periodontal disease [8] that is defined as the deviation of tooth position due to force, inflammation, or both. The prevalence of pathological tooth migration ranges from 30.3 to 55.8%. Common etiologies include periodontal tissue destruction, occlusal forces, soft tissue pressure, oral habits, and gingival enlargement [8–11]. Esthetics and proper occlusal function are compromised by pathological tooth migration. Treatment should begin with diagnosis to identify the etiological factors (e.g., tissue inflammation, loss of support, occlusal interferences); eliminating these factors could results in complete or partial resolution of the malalignment [12].

The case presented in this paper details the treatment process of an immunosuppressed renal transplant receiver with complex conditions including severe periodontitis, gingival enlargement, and pathological tooth migration.

## Case presentation

A 51-year-old woman presented with chief complaints of bleeding gums and chewing disability. Her medical history revealed that she had received renal transplantation surgery in 1999 and had remained in a stable condition without graft-versus-host disease ever since. She suffered from hypertension and gout, both of which were being well controlled and followed by treating physicians. Her medications at the time of initial examination including the immunosuppressive regimen (cyclosporine, prednisolone, and mycophenolate mofetil) are listed in Table 1. The patient was a nonsmoker who did not consume alcohol. She stated that she seldom brushed her teeth and had not flossed for a long time because of serious gum bleeding upon brushing or flossing.

**Table 1:** Medications at initial examination

Medication	Usage (Class)	Dosage
Cyclosporine	Immunosuppressive (Calcineurin inhibitor)	50 mg/BID
Prednisolone	Immunosuppressive (Corticosteroid)	5 mg/QD
Mycophenolate mofetil	Immunosuppressive (Anti-proliferative)	250 mg/BID
Atenolol	Blood pressure control (β-blocker)	100 mg/QD
Benzbromarone	Gout treatment (Uricosuric agent)	50 mg/QOD
Colchicine	Gout treatment (Anti-inflammatory)	0.5 mg/QOD

**Fig. 1:**
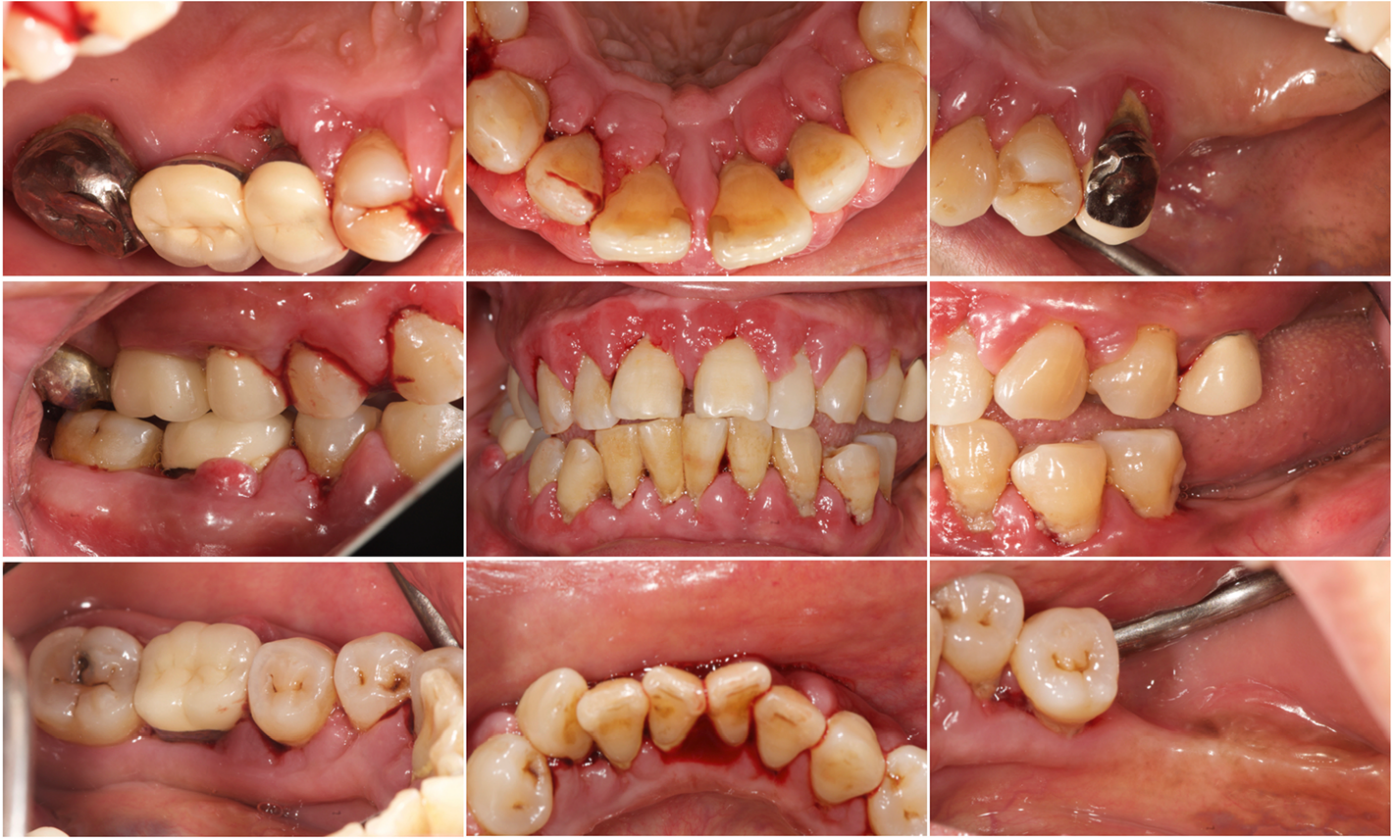
Initial clinical views. Inflammatory and fibrotic gingivae with spontaneous bleeding tendency. Note a 2.5-mm diastema between the upper central incisors, fistula over the lower right first molar, and malocclusion over the left premolars due to hypermobility

**Fig. 2:**
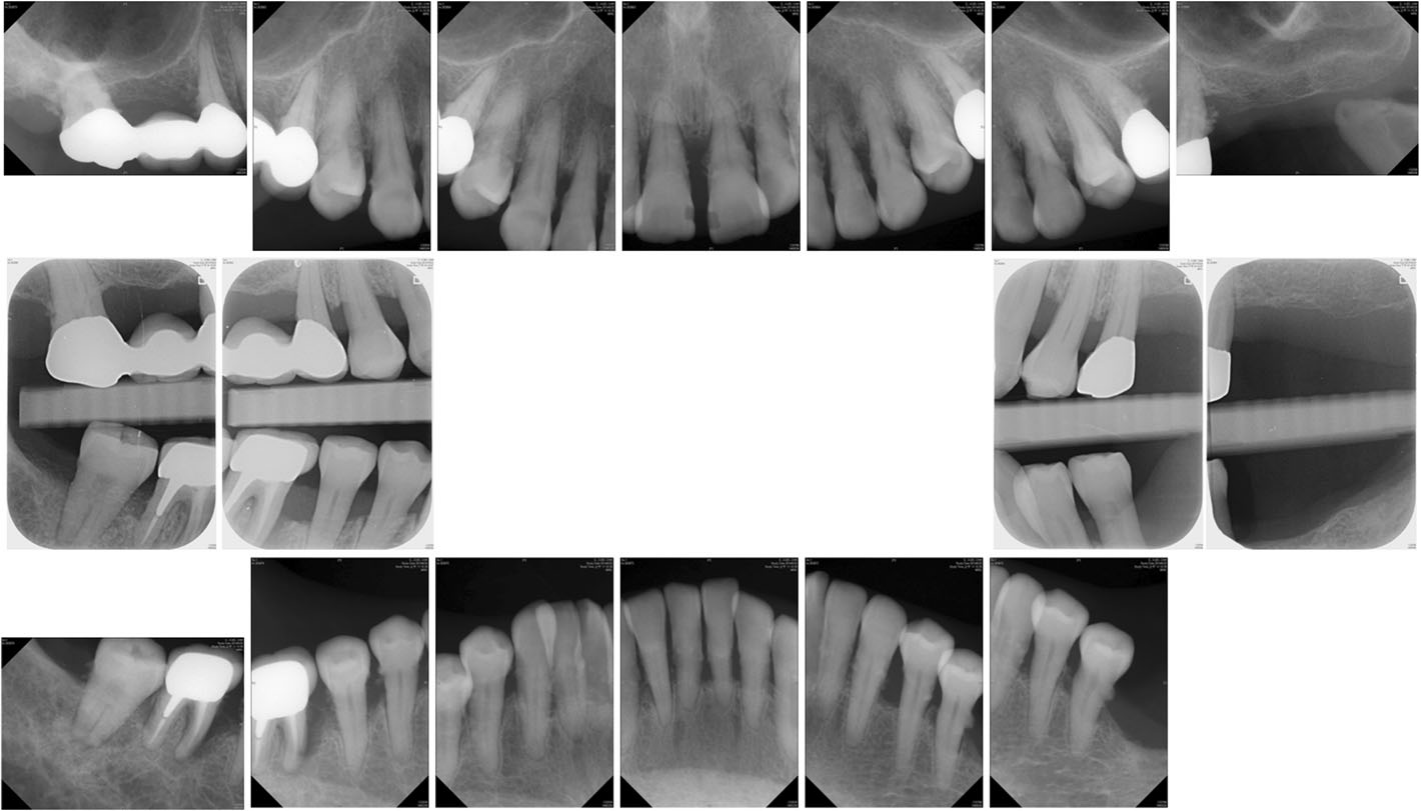
Initial radiographs. Note the bone loss over mesial root of the lower right first molar and multiple subgingival deposits

A facial examination revealed normal appearance without lymph node enlargement or local heat. No temporomandibular joint related symptoms were reported. Normal hard and soft tissue were observed in the patient’s oropharynx, hard and soft palates, floor of the mouth and tongue. An intraoral examination revealed highly inflamed periodontal tissue with heavy plaque and calculus deposition. Fibrotic changes over interdental papillae, facial and also lingual gingivae were noted, especially in the anterior segment (Fig. 1). The patient’s upper left and lower left molars were missing. Her anterior teeth were flaring and highly mobile with a diastema of approximately 2.5 mm between the maxillary central incisors. Upon examination, we noted full mouth deep probing pocket depth (up to 15 mm) and profound bleeding. A fistula with suppuration was observed over buccal gingiva of tooth 46 (lower right first molar). Periapical films revealed generalized horizontal bone loss with the supporting bone of less than one half or one third of the root length. Periradicular radiolucency with an angular bony defect extending beyond the apex was observed over the mesial root of tooth 46 (Fig. 2).

The clinical diagnosis was severe periodontitis and gingival disease modified by medication [13]. A hopeless prognosis was assigned to tooth 46. After consultation with treating physicians and discussion with the patient, a tentative treatment plan was formed involving cause-related periodontal treatment and extraction of tooth 46. In addition, the immunosuppressive regiment was not altered.

**Fig. 3:**
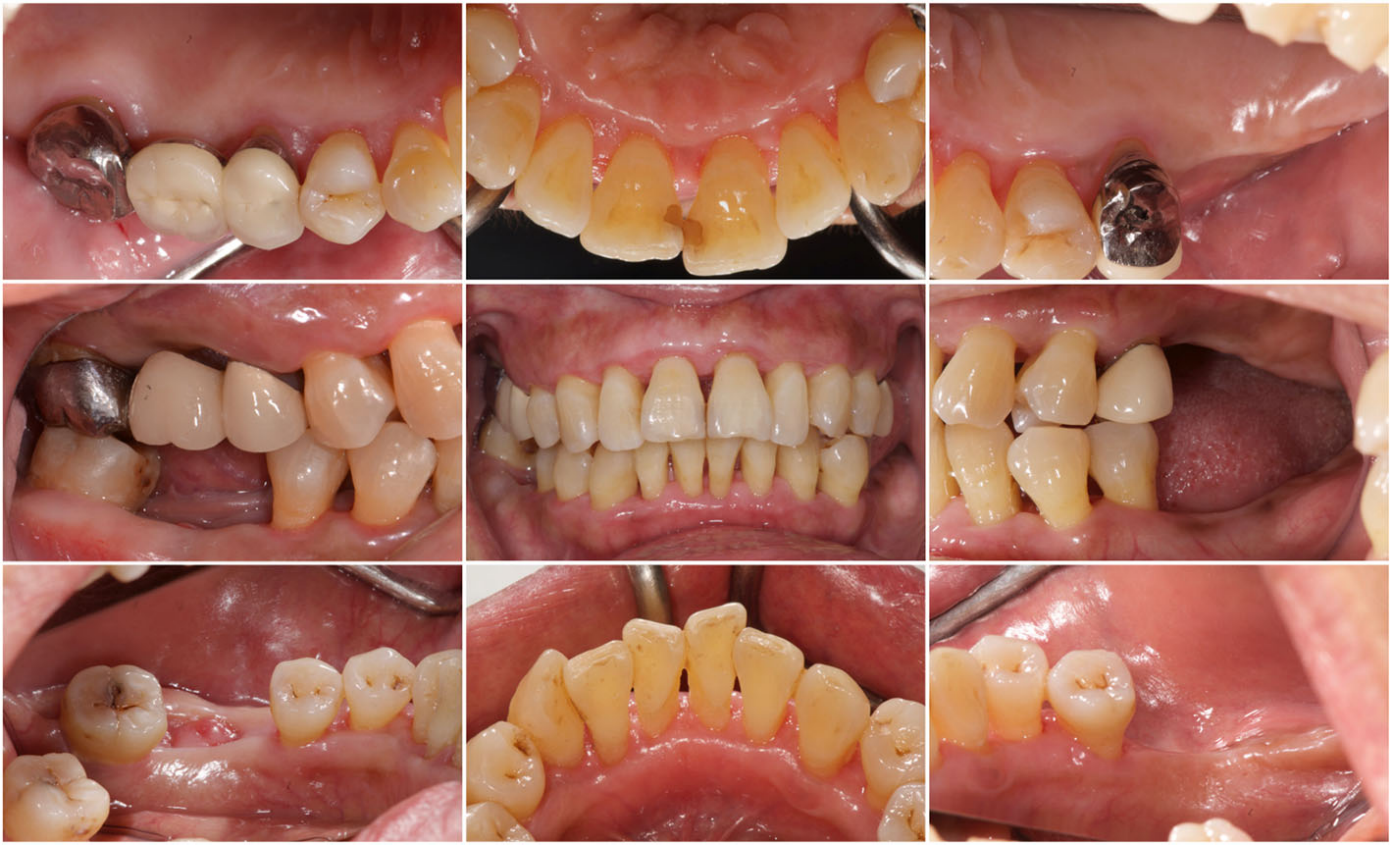
Re-evaluation clinical views. The gingivae were less edematous and glazed. The diastema had closed. The extraction wound over the lower right first molar was in the process of healing. Occlusal contact had been regained over the left premolars

The patient was treated in the Department of Periodontics in Taipei Chang Gung Memorial Hospital between July 2014 and January 2015. Dental appointments were coordinated with physician appointments to obtain blood test results before each dental visit. Data were mostly within the normal range except for the occurrence of mild thrombocytopenia (listed in Table 2). Prophylactic antibiotics (amoxicillin, 2 g) were prescribed before each appointment [14]. The treatments included scaling, root planing, oral hygiene instruction, and extraction. Although tooth mobility decreased after multiple visits, we discovered occlusal interference during the patient’s lateral excursion and anterior jaw movement; therefore, occlusal adjustment was incorporated into the treatment plan. Six months after the patient’s initial visit, a marked improvement of her periodontal condition as well as more effective plaque control were observed. The gingivae were considerably less edematous and glazed and without any residual fibrotic appearance (Fig. 3). The periodontal probing result revealed some residual but largely decreased periodontal pockets and grade I tooth mobility over the upper and lower anterior teeth and lower right second molar [15]. In addition, the diastema between the upper incisors closed “spontaneously” after treatment.

**Table 2:** Blood test results prior to each appointment

Test/visit	Treament1	Treament2	Treament3	Treatment4	Treatmen5	Treatment6	Extraction
eGFR (mL/min/1.73)	44	40	41	41	41	33	44
Creatinine (mg/dL)	1.29	1.39	1.36	1.35	1.37	1.64 (high)	1.27
RBC (10^6^/uL)	3.84	3.81	3.72	3.78	3.77	3.72	3.7
Hemoglobin (g/dL)	12.4	12.1	11.9 (low)	12	12.2	11.8 (low)	11.7 (low)
Hematocrit (%)	37.2	36.8	35.6 (low)	36.7	36.6	36.5	36.2
WBC (10^3^/uL)	5.8	7.2	5.3	4.7	6.1	6.2	5
Platelet (10^3^/uL)	103 (low)	87 (low)	96 (low)	101 (low)	111 (low)	102 (low)	112 (low)

**Fig. 4:**
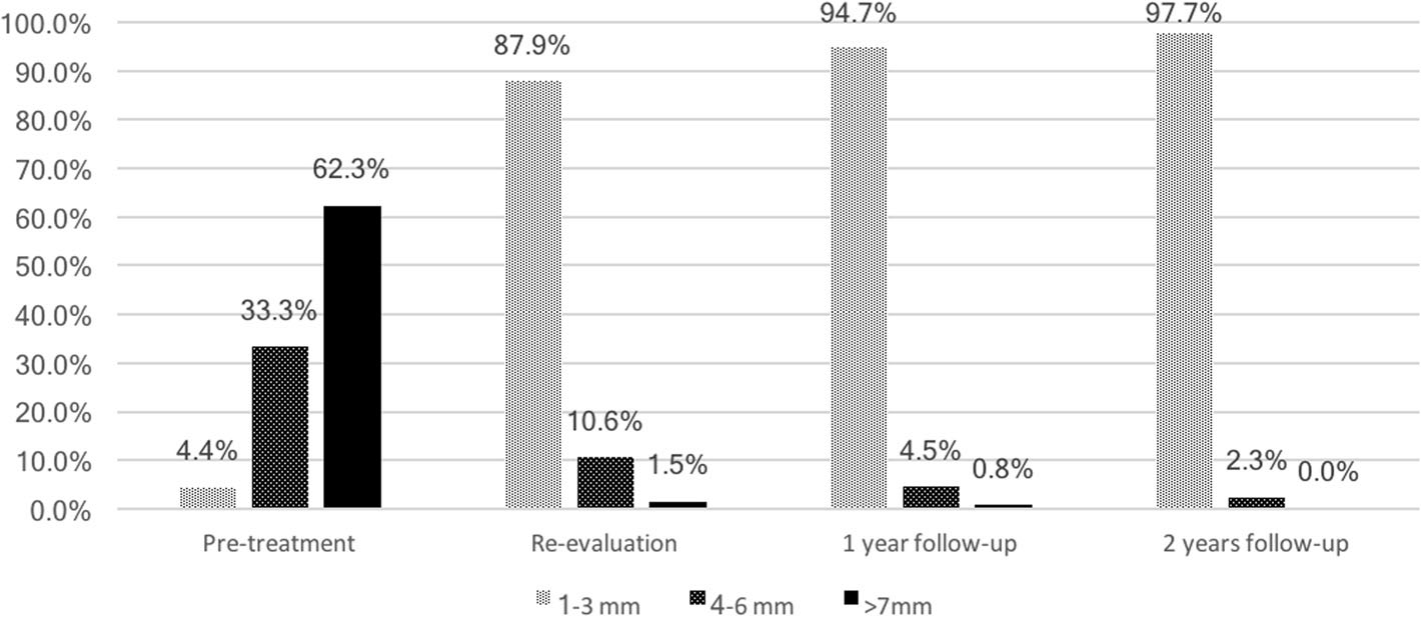
Probing pocket depth distribution and changes

**Fig. 5:**
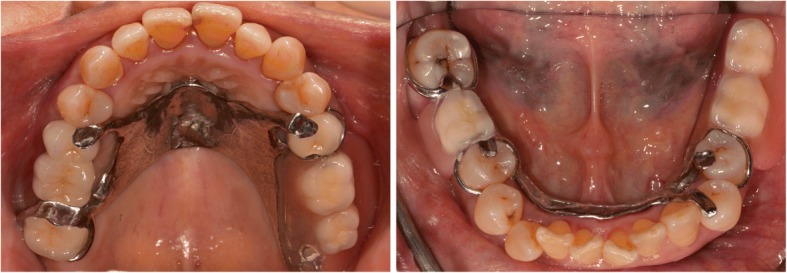
Rehabilitation with removable partial dentures. The palatal strap and lingual bar with wrought-wire design favored periodontal compromised abutment teeth

**Fig. 6:**
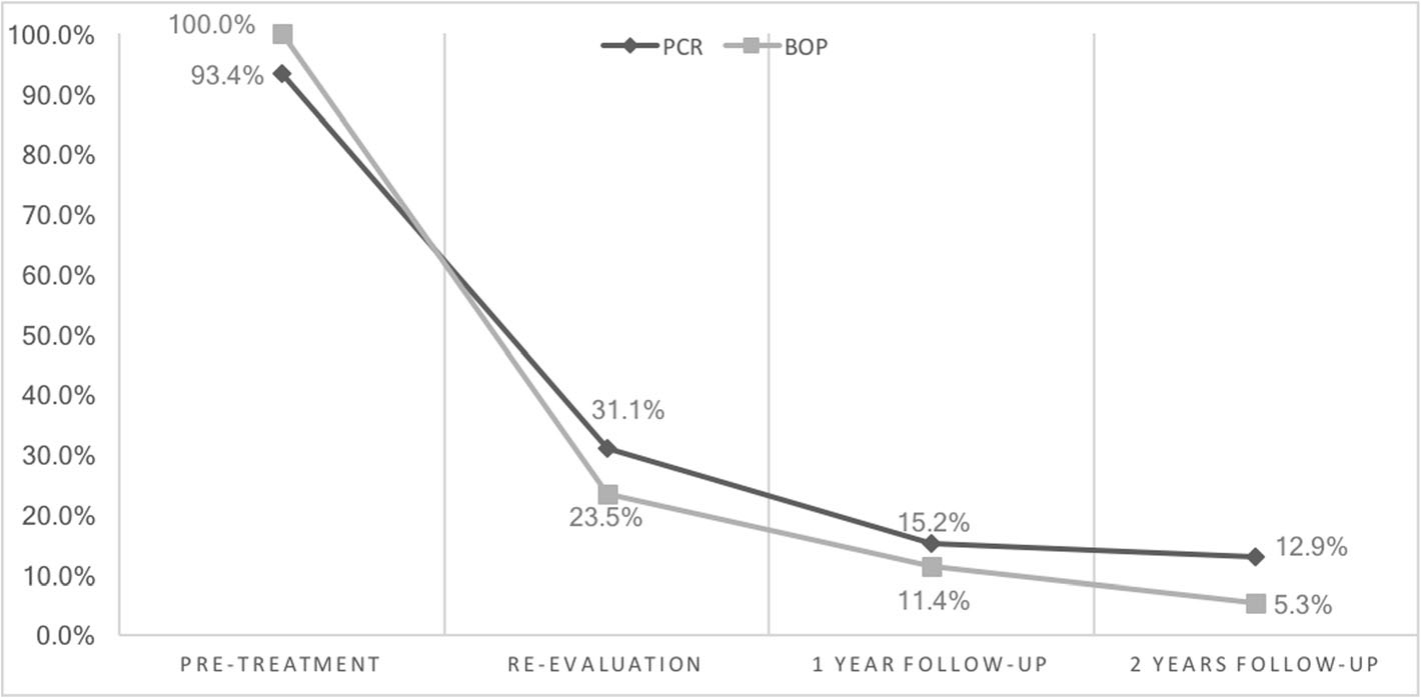
Plaque control record (PCR) and bleeding on probing (BOP) distribution and changes

**Fig. 7:**
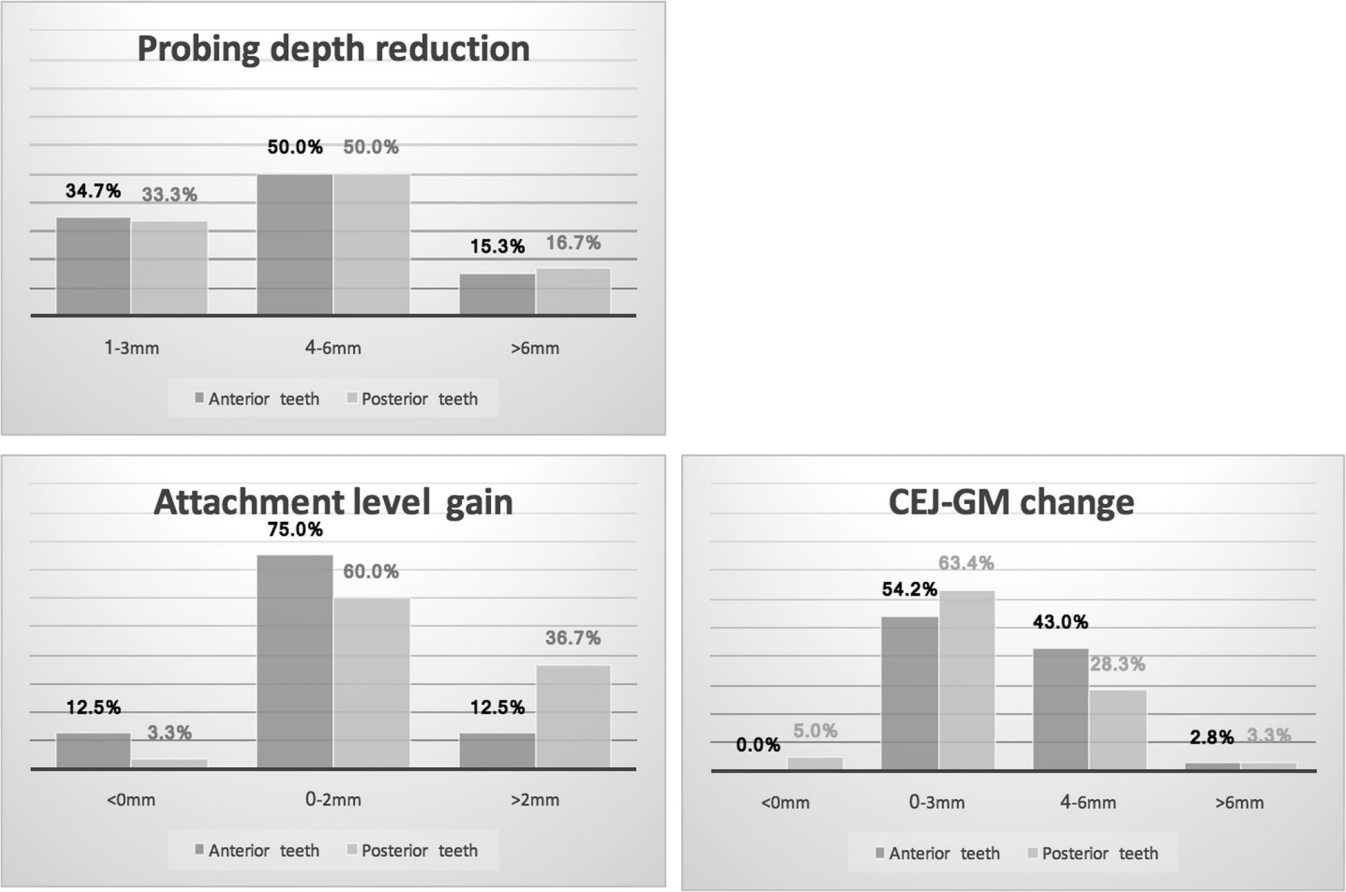
Clinical probing data distribution and changes, comparison between anterior and posterior teeth

**Fig. 8:**
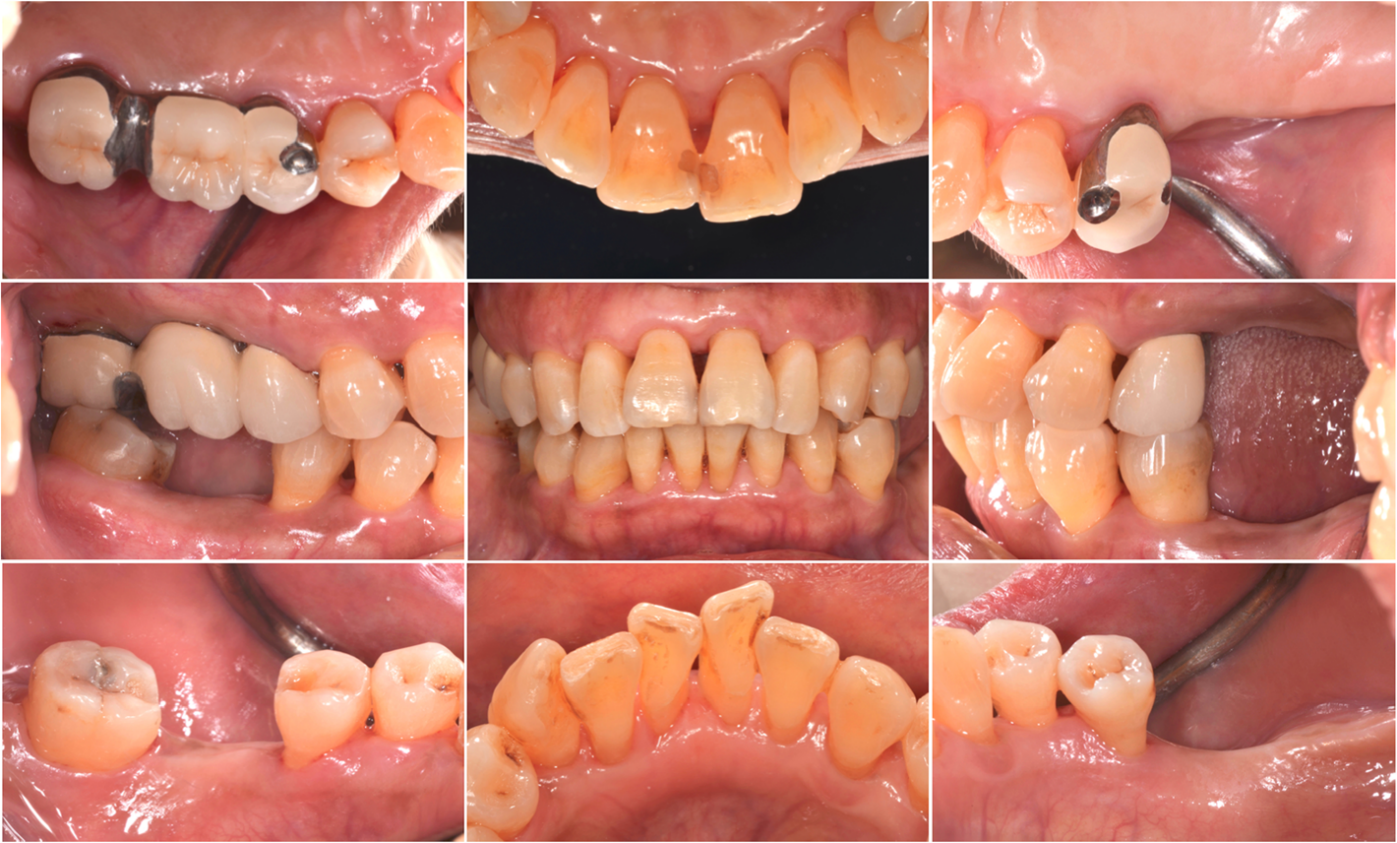
Two-year follow-up clinical views. Stable tissue tonus and effective plaque control performed by the patient. The diastema had remained closed. A healed socket can be observed over the lower right first molar site

**Fig. 9:**
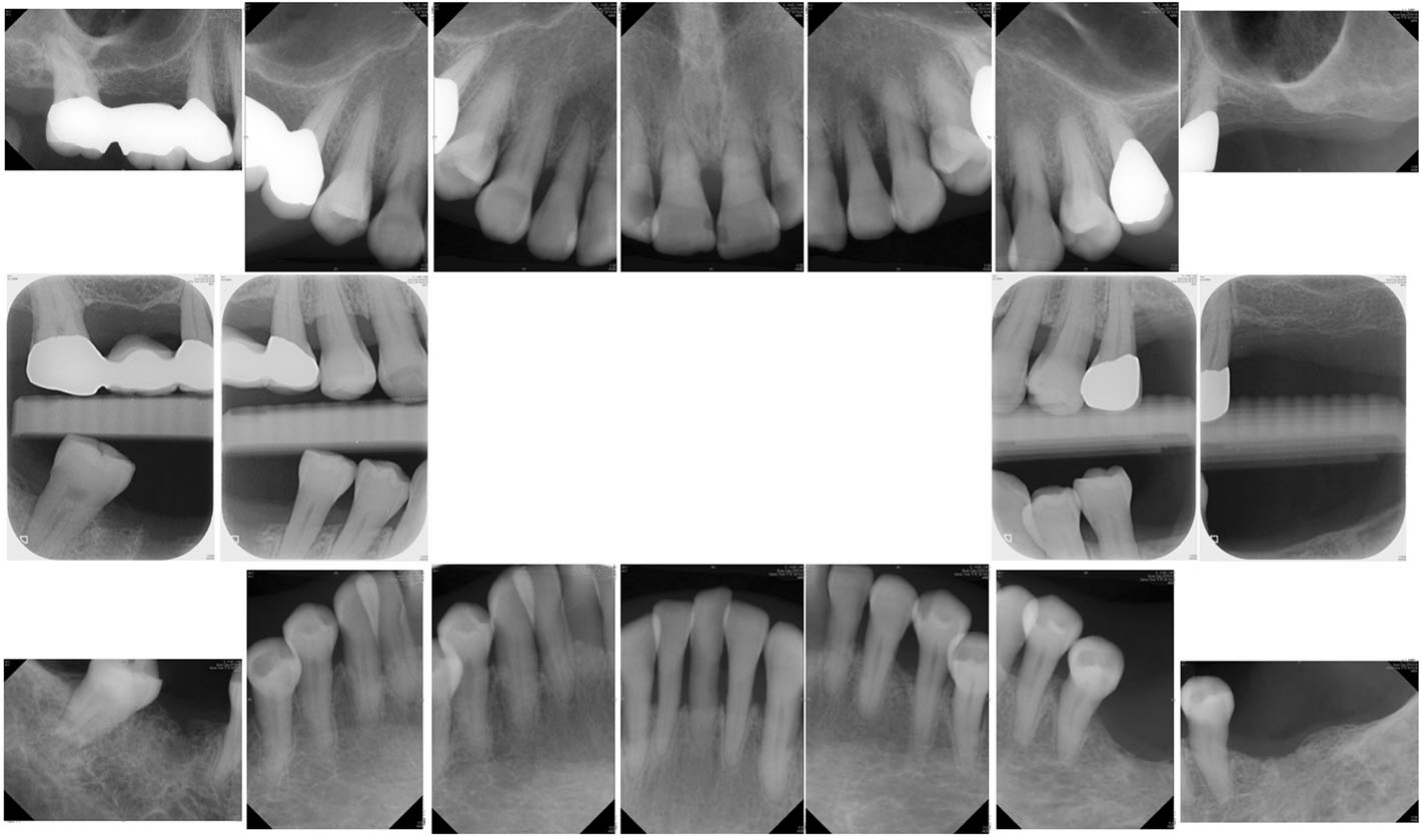
Two-year follow-up radiographs. The bone level was maintained throughout the treatment course

Pocket elimination periodontal surgery was initially planned to treat the upper right and lower right second molars; however, nonsurgical treatment was eventually selected as the final treatment plan after the patient rejected surgery and the residual periodontal pocket depth were mostly less than 6 mm with horizontal bone loss pattern (Fig. 4) [16]. After 3 months of follow-up, the patient was referred to our prosthodontic department for oral rehabilitation with removable partial dentures. The dentures were designed with palatal strap, lingual bar, and wrought-wire clasps (Fig. 5). The periodontal supportive treatment recall interval was monthly in the first year and every 3 months subsequently [17]. A clinical examination and periodontal probing after 2 years of follow-up revealed excellent oral hygiene, continuous pocket reduction, and tooth mobility within normal limit for all remaining teeth (Figs. 4 and 6). The difference of attachment level gain, probing depth reduction, and change of the distance from cementoenamel junction to gingival margin (CEJ-GM) between anterior (canine to canine) and posterior teeth were shown in Fig. 7. No recurrence of gingival enlargement was recorded throughout the follow-up period. The diastema between the upper incisors remained closed without centric or lateral occlusal interference (Fig. 8). Full mouth periapical and bite-wing radiographs revealed that the alveolar bone level had been maintained from its initial condition (Fig. 9). The treating prosthodontist also recalled the patient regularly. The patient reported good chewing function, considerable ease in conducting oral hygiene practices, and overall high satisfaction with the treatment outcomes.

## Discussion and conclusions

The main focus of nonsurgical periodontal treatment is to reduce tissue inflammation by eliminating bacterial biofilm, and the key to a successful outcome is the thoroughness of root debridement and patients’ own plaque control ability [18]. According to previous studies, the overgrown tissue induced by cyclosporine tends to be more inflammatory and less fibrotic in nature than that induced by other agents [19, 20]. By understanding these histological factors, we can reasonably assume that to some extent, such patients are less likely to require surgery. Nonsurgical periodontal therapy includes scaling, root planing, and oral hygiene reinforcement, were performed meticulously over multiple appointments in the case presented in this study; thus, the evident reduction in tissue enlargement was anticipated.

Although no precise dental treatment protocol for renal transplant patients has been established, the clinical approach should focus on eliminating infection sources and creating an oral environment that facilitates easy plaque control. Good communication between dentists and treating physicians is also very important. Practically, a treatment course should include oral hygiene instruction, use of antimicrobial mouthwash, scaling, root planing, and extraction if necessary. Antimicrobial prophylaxis in accordance with the American Heart Association’s regimen before invasive treatment was suggested; however, no evidence-based research supports the effect of it [14, 21]. Clinicians should check patients’ bleeding tendencies before invasive procedures, especially the platelet count [22, 23]. Furthermore, specific medications, such as nonsteroidal anti-inflammatory drugs, macrolide antibiotics, and aspirin, should be avoided because they may interact with cyclosporine and raise the serum level of it due to patients’ impaired renal function [24]. Long-term corticosteroid intake (as a supplementary immunosuppressive agent) may induce adrenal crises during pressure events such as prolonged and invasive dental treatment. Although avoidance of supplementary steroidal coverage was recommended in a double-blind study [25], patients should be administered with a stress-relieving protocol, and their blood pressure should be monitored during treatment [26].

Specific oral manifestations have been observed in renal transplant patients. In a descriptive study regarding clinical oral findings in patients who had undergone kidney transplants, the most prevalent finding was saburral tongue, followed by xerostomia, gingival enlargement, and oral candidiasis [27]. Various infectious lesions including bacterial, viral, and fungal lesions were observed in immunosuppressed patients [26]. Incidence of non-melanoma skin cancer was reported as 20 to 40 times higher in renal transplant receivers [28]. If such lesions are suspected, screening via culture or biopsy should be performed.

Although the detailed mechanism underlying cyclosporine-induced gingival enlargement is not fully known, interactions between the drug and genetically-determined susceptible gingival fibroblasts are widely believed to enhance protein synthesis, collagen production, and cell proliferation. In addition, elevated inflammatory cytokines (e.g., interleukin [IL]-6, IL-1β) upregulate protein synthesis, which, combined with the decreased secretion of collagenase (e.g., matrix metalloproteinase [MMP]-1, MMP-3), results in tissue proliferation [4, 29]. Many risk factors for cyclosporine-induced gingival enlargement have been identified, including gender (male with higher risk), young age, high drug dosage, concomitant calcium channel blockers, impaired renal function [30], certain human leukocyte antigen phenotypes [31], and the presence of bacterial plaque [32].

For clinicians, the first approach to treat patients with immunosuppression and complex dental conditions should be consulting the treating physician in order to fully understand a patient’s current systemic condition. Subsequently, plaque-induced tissue inflammation should be eliminated by performing thorough cause-related therapy, and high-quality plaque control performance should be demanded of the patient. The literature contains many reports of resolving gingival enlargement or reducing the need for surgery through plaque control programs alone; most such articles have reported significant improvements in more than half of the patients in their study cohorts [33, 34]. Also, use of an adjunctive antiseptic mouthwash (chlorhexidine 0.1–0.2%) might reduce the severity of enlargement and prevent its recurrence [35].

If tissue inflammation persists after meticulous nonsurgical debridement or the enlarged tissue is impairing a patient’s periodontal health, self-performed cleaning ability, or esthetic appearance, surgical intervention is indicated. Many approaches for removing excessive tissue are available, including scalpel gingivectomy, flap surgery, electrosurgery, and laser gingivectomy. In one study, the application of laser yielded a lower recurrence rate and higher patient satisfaction [36]. Switching a patient’s immunosuppressive medication to tacrolimus could effectively resolve or in some cases completely reverse the gingival condition without surgical intervention [37, 38]. Consultation with a treating physician is required for drug alteration; however, such alterations are sometimes impossible because of medical or financial concerns [39].

By comparing the clinical data of anterior and posterior teeth from initial to 2-year follow up, it can be recognized, while the difference in probing depth reduction is minor, the attachment level gain was more pronounced in posterior segment, and the CEJ-GM level change (“gingival recession”) was greater in the anterior teeth (Fig. 7). This is in accordance with the literature [4] and the initial clinical view of the current case report (Fig. 1), that the greatest amount of tissue enlargement was noted in the anterior segment. In the current case report, gingival enlargement gradually resolved throughout the course of a 6-month meticulous plaque control program without surgical intervention nor change in medication, and maintained in stable condition for more than 2 years (Figs. 4 and 6). Understanding the histology of the affected tissue [20], detailed diagnosis based on periodontal probing with radiographs depicting the bone loss pattern, and meticulous treatment and a short follow-up interval are keys to the success of conservative treatment approaches.

Periodontal treatment alone has the potential to “spontaneously” correct pathologically migrated tooth positioning [12, 40]; however, frequency and predictability are seldom mentioned in the literature. In a prospective clinical study that analyzed 33 periodontal patients with secondary diastemata, 36.4% and 51.5% of all tooth gaps were completely closed after nonsurgical periodontal treatment and surgical debridement, respectively. Moreover, the frequency was higher when the diastema was less than 1 mm [41]. In summary, according to the literature, elimination of periodontal tissue inflammation and minor occlusal adjustment should constitute the preliminary approach to treating pathologically migrated teeth. After inflammation and unfavorable forces have been controlled, orthodontic alignment or prosthodontic intervention can close the residual space and restore a patient’s previous esthetic appearance.

In the patient investigated in the present study, the spacing between the upper anterior teeth closed and remained stable throughout the follow-up period after inflammation had been controlled and occlusal interferences had been eliminated. Prosthetic rehabilitation to regain posterior support facilitated long-term stability. No orthodontics were incorporated into the treatment process.

In conclusion, medically compromised patients with multiple dental problems remain a clinical challenge. Clinicians must fully understand patients’ systemic conditions and consider every potential treatment option before making decisions. With proper diagnosis, treatment planning, and patient cooperation, complex conditions can be managed through simple approaches.

## Abbreviations

CEJ-GM: CementoEnamel Junction to Gingival Margin
CKD: Chronic kidney disease
ESRD: End stage renal disease
IL: Interleukin
MMP: Matrix metalloproteinase
